# Retraction Note: Online learning challenges in Thailand and strategies to overcome the challenges from the students’ perspectives

**DOI:** 10.1007/s10639-026-13962-w

**Published:** 2026-03-14

**Authors:** Sayam Aroonsrimarakot, Meena Laiphrakpam, Pokkasina Chathiphot, Prayoon Saengsai, Sirorat Prasri

**Affiliations:** 1 Interdisciplinary Research and Development Committee, Royal Society of Thailand, Bangkok, Thailand; 2 Center for Research Assessment and Certification of Environmental Management, Faculty of Environment and Resource Studies, Mahidol University, Salaya Nakhon Pathom, Thailand; 3 Sakhon Nakhon Rajabhat University, Sakon Nakhon, Thailand; 4 Mahachulalorngkornrajavidyalaya University, Khonkaen, Thailand

**Retraction Note: Education and Information Technologies (2022) 28:8153–8170**

10.1007/s10639-022-11530-6

The Editor-in-Chief has retracted this article because prospective ethics approval was not obtained prior to the commencement of the study.

Meena Laiphrakpam has not stated whether they agree or disagree with this retraction. Sayam Aroonsrimarakot, Pokkasina Chathiphot, Prayoon Saengsai, and Sirorat Prasri have not responded to correspondence regarding this retraction.

